# EARLY DETECTION OF COGNITIVE IMPAIRMENT IN PRIMARY CARE: IMPLEMENTATION CHALLENGES AND OPPORTUNITIES

**DOI:** 10.1093/geroni/igae098.2846

**Published:** 2024-12-31

**Authors:** Amy Deckert, Laura Chavira, Katherine Selzler, Karen Weyrauch, James Murray, Alissa Kurzman, Tim MacLeod

**Affiliations:** Davos Alzheimer’s Collaborative, Toronto, Ontario, Canada; Davos Alzheimer’s Collaborative, Wayne, Pennsylvania, United States; Davos Alzheimer’s Collaborative, Wayne, Pennsylvania, United States; Davos Alzheimer’s Collaborative, Wayne, Pennsylvania, United States; Davos Alzheimer’s Collaborative, Wayne, Pennsylvania, United States; Davos Alzheimer’s Collaborative, Wayne, Pennsylvania, United States; Davos Alzheimer’s Collaborative, Wayne, Pennsylvania, United States

## Abstract

Healthcare systems worldwide are not prepared to adequately address the needs of the 55 million individuals affected by Alzheimer’s and related dementias. A substantial 17 to 20-year lag exists between development of innovations and their widespread adoption, with less than 50% being used in clinical practice (Bauer et al. 2020). In the early stages of Alzheimer’s disease, cognitive impairment often goes unrecognized until later in the disease course, and it can take months or years to reach a diagnosis. Early detection in primary care is a pivotal factor in improving patient outcomes and ensuring timely access to appropriate care and interventions. The Davos Alzheimer’s Collaborative Healthcare System Preparedness (DAC-SP) Early Detection flagship program implemented digital cognitive assessments (DCA) and a blood-based biomarker (BBM) test in diverse primary care or other non-specialty settings across 7 healthcare systems in 6 countries (Brazil, Jamaica, Japan, Mexico, Scotland, US). We conducted a mixed methods evaluation of the process and outcomes of the DAC-SP flagship study guided by the Consolidated Framework for Implementation Research (CFIR; Damschroder et al., 2009) and report here the qualitative findings from the pre-implementation phase. We employed a directive content analysis of transcribed in-depth interviews with clinicians (n=51) and implementation team leads (n=7) across flagship sites. Our findings underscore the critical importance of addressing system-level factors in the design and implementation of DCA and BBM tests in primary and non-specialty care, suggesting that sustainable practice change requires a need to shift the focus from individual to systemic interventions.

